# An audit of airway complications in a district general hospital ICU

**DOI:** 10.1186/cc10746

**Published:** 2012-03-20

**Authors:** JW Chan, KJ Turner, R Lloyd, R Howard-Griffin

**Affiliations:** 1 Ipswich Hospital NHS Trust, Ipswich, UK

## Introduction

The 4th National Audit Project of The Royal College of Anaesthetists and The Difficult Airway Society (NAP4) highlighted the increased incidence of airway-related complications in an ICU setting [1]. The aim of this audit was to establish our local ICU airway intubation complication rate as well as our compliance with the NAP4 recommendation that continuous capnography should be used on all intubated patients.

## Methods

All intubated patients who were admitted to the Ipswich Hospital ICU between April and December 2010 were identified and data relating to basic demographics, airway management and the use of capnography were collected. An airway was classed as difficult if there were two or more attempts at intubation, a bougie was used, or it was Cormack-Lehane grade III/IV. Complications arising from airway intubation were also noted.

## Results

A total of 139 intubations on 118 patients were identified. Fifty-eight (42%) intubations occurred on the ICU, 41 (29%) in the emergency department (ED) and five (4%) on the ward; 29 (21%) intubations occurred in theatre for surgery and six (4%) out of hospital. Of the 104 intubations on the ICU, ED or ward, nine (9%) were classed as difficult and there were 21 (20%) documented complications (hypoxia, hypotension, oesophageal intubation, cardiac arrest and aspiration). Complication rates were similar across junior trainees, senior trainees and consultants. Only 27% of all intubated patients received continuous capnography.

## Conclusion

Our findings are consistent with the NAP4 view that airway management outside the controlled confines of a theatre setting has the potential to be more difficult. Steps should be taken to minimise the risk associated with this procedure, including a thorough airway assessment, use of continuous capnography and the presence of suitably trained operators and assistants. The finding that complications occurred at a similar rate regardless of the seniority could be explained by more senior staff intubating the most unwell patients.
